# Emerging therapeutic agents for advanced non-small cell lung cancer

**DOI:** 10.1186/s13045-020-00881-7

**Published:** 2020-05-24

**Authors:** Ruqin Chen, Rami Manochakian, Lauren James, Abdel-Ghani Azzouqa, Huashan Shi, Yan Zhang, Yujie Zhao, Kexun Zhou, Yanyan Lou

**Affiliations:** Division of Hematology and Oncology, Mayo Clinic, Jacksonville, FL 32224 USA

**Keywords:** Advanced non-small cell lung cancer, NSCLC, Emerging therapeutic agents, Targeted therapy, Immunotherapy, Clinical trials

## Abstract

Advanced non-small cell lung cancer (NSCLC) is the most common type of lung cancer, with a poor prognosis and no known cure. Survival time is often short because of limited treatment options. Recent advances in targeted therapy and immunotherapy have changed the landscape for the treatment of advanced NSCLC. In the last 10 years, the US Food and Drug Administration (FDA) has approved more than 17 new medications for this devastating disease and more are coming. Molecular and immunogenic testing makes personalized medicine possible for patients with advanced NSCLC. The new medications provide promising efficacy and safety resulting in improved long-term survival for a significant number of patients. In this review, we summarize the recent advances in advanced/metastatic NSCLC therapeutics with a specific focus on first in-human or early-phase I/II clinical trials. These drugs either offer better alternatives to current standard drugs in the same class or are a completely new class of drugs with novel mechanisms of action. Advances are divided into (1) targeted agents, (2) antibody-drug conjugates, and (3) immunotherapies. Finally, we present a brief review of the emerging agents and ongoing clinical studies.

## Introduction

Lung cancer is the most common cause of cancer-related death and the second most common malignancy reported in the USA and worldwide. It is estimated that in 2020 there will be 228,820 newly diagnosed cases of lung cancer and 135,720 deaths attributed to lung cancer. The total number of deaths attributed to lung cancer is greater than from colon, prostate, and breast cancer combined. This dismal outcome in lung cancers is due, in part to the fact that more than half of the patients, about 55%, presented with metastatic lung cancer at the time of diagnosis [1]. Finally, non-small cell lung cancer (NSCLC) comprised about 85% of the newly diagnosed lung cancer cases. Advanced NSCLC includes those who present with metastatic disease or recur following initial definitive treatment.

Median overall survival (OS) for metastatic NSCLC patients is about 4–5 months with supportive care alone. For patients that receive supportive care in conjunction with induction platinum-based chemotherapy, historically, the median OS has been 8–12 months. For decades, multiple trials have compared different chemotherapy regimens and resulted in marginal improvements in the OS [2, 3]. Research examining the treatment benefits of chemotherapy has plateaued. In 2002, the Eastern Cooperative Oncology Group published results of a randomized phase III trial comparing four platinum-based doublets in first-line metastatic NSCLC. The trial demonstrated no difference in overall survival among the different treatment regimens.

In 2004, a randomized phase II trial comparing chemotherapy versus bevacizumab plus chemotherapy reported results that showed that NSCLC patients with non-squamous type responded better to bevacizumab with chemotherapy. Similarly, the impact of histology to treatment was also seen with pemetrexed. Pemetrexed was shown to only be effective in non-squamous cell carcinoma. Finally, results from a phase 3 trial examining platinum-based chemotherapy followed by maintenance pemetrexed showed a median OS rate of 13.9 months, as compared with 11 months among patients randomly assigned to supportive care alone after induction chemotherapy. Median OS from induction in patients received pemetrexed was 16.9 months compared to 14 months in patients without supportive care alone [4–6].

A major advancement in the treatment of metastatic NSCLC came with the identification of specific driver mutations and the development of targeted therapy. Although the subset of patients with actionable mutations is small, progression-free survival was shown to be significantly increased in patients treated with targeted therapy compared to those treated with chemotherapy. The response rate range is 50 to 80% for patients who harbored EGFR, ALK, ROS1, and BRAF mutations and received targeted therapy. Overall survival was increased to between 18 and 38.6 months [7–9].

The development of immune checkpoint inhibitors was the next breakthrough in the treatment of metastatic NSCLC. Research has shown that inhibitors of programmed death 1 (PD-1) and the ligand PD-L1 are effective in metastatic NSCLC as first-line and second-line treatment options. Nivolumab, an immune checkpoint inhibitor, was the first approved second-line treatment in immunotherapy for metastatic NSCLC. Nivolumab, when compared with docetaxel, improved the median OS in squamous and non-squamous NSCLC. Additionally, pembrolizumab was approved as a single agent for first-line treatment and showed a higher OS rate at 6 months than chemotherapy alone among patients with high PD-L1 level > 50% who did not have targetable mutations.

Recent studies demonstrate that pembrolizumab in combination with chemotherapy improved the OS irrespective of PD-L1 status when compared to chemotherapy alone; in both non-squamous and squamous histology types. For patients with non-squamous subtype, receiving pembrolizumab in combination with chemotherapy, the hazard ratio for death was 0.49 with a 12-month OS rate of 69.2%. For patients with the squamous subtype, who received pembrolizumab-chemotherapy combination, the hazard ratio for death was 0.64 with a median OS of 15.9 months. Before the introduction of immunotherapy with checkpoint inhibitors, the 5-year survival rate for patients with advanced NSCLC was 4–6%. Long-term survival significantly improved with the addition of immunotherapy. A phase I trial examining the efficacy of nivolumab as a second-line treatment, resulted in increasing the estimated 5-year overall survival rate to 16% [10]. In another phase I trial, examining the efficacy of pembrolizumab, the 5-year overall survival was 23.3% in first-line setting and 15.5% in the second-line setting. Among patients with a PD-L1 > 50% treated with pembrolizumab, 5-year overall survival is more than 25% in both first- and second-line setting [11].

In the past decade, treatments for advanced NSCLC have dramatically evolved and enabled more individualized selection of treatment options. Molecular profiles and immunologic status help to determine treatment options. For example, patients who harbor EGFR mutations, ALK rearrangement, ROS1 rearrangement, BRAF mutation, NTRK mutation, and high PD-L1 level should have FDA-approved targeted therapy or immunotherapy as first line. Other oncologic driver mutations such as RET, MET, and HER2 in NSCLC are also promising targets for treatment. Immunologic blockades to PD-1, PD-L1, and CTLA-4 are also blooming.

This paper reviews recently published data on some of the most promising lines of treatment. We reviewed currently approved and emerging systemic therapies for advanced NSCLC. A literature search was done in PubMed, Google Scholar, ClinicalTrial.gov, American Society of Clinical Oncology (ASCO) meeting abstracts, World Conference on Lung Cancer (WCLC) abstracts, and American Association for Cancer Research (AACR) abstracts to identify phase 1/2, first in human clinical trials in advanced/metastatic non-small cell lung cancer. Each study was individually reviewed and data points have been summarized (Table 6).

## Targeted therapy

### Epidermal growth factor receptor (EGFR) tyrosine kinase inhibitors (TKIs)

EGFR belongs to a family of receptor tyrosine kinase that includes EGFR/ERBB1, HER2/ERBB2/NEU, HER3/ERBB3, and HER4/ERBB4.

**Table 1:** FDA-approved EGFR TKIs

Drug	Month/year	Indication	Comments
Erlotinib	May/2013	1st line metastatic EGFR mutant	First generation. Approved 18 November 2004 for 2nd-line metastatic NSCLC. 10 April 2010 maintenance
Gefitinib	July/2015	1st line metastatic EGFR mutant patients	Approved in 2003 as third line, retracted approval 2005
Afatinib	July/2013	1st line metastatic EGFR mutant	Second generation
	January/2018	1st line metastatic EGFR non-resistant mutations	
Osimertinib	November/2015	EGFR T790M mutation after progressed on EGFR TKI	Third generation. With CNS penetration
	April/2018	1st line metastatic EGFR mutant patients	
Dacomitinib	September/2018	1st line metastatic EGFR mutant patients	Second generation

EGFR mutations are present in 15% of patients with NSCLC in the Western population and rising to 35% in the Asian population. Additionally, there is a higher prevalence of EGFR mutations in never smokers. These mutations occur within EGFR exons 18–21 and about 90% of these mutations are exon 19 deletions or exon 21 L858R point mutations, which refer to EGFR mutant as above. These mutations are sensitive to EGFR TKIs. EGFR mutations on exon 18 and exon 20 are usually less sensitive to EGFR TKIs.

Gefitinib is the first tested EGFR inhibitor in advanced NSCLC. Iressa Pan-Asia Study (IPASS) reported progression-free survival was significantly longer among those who received gefitinib than among those who received chemotherapy (hazard ratio for progression or death, 0.48). However, erlotinib is the first approved in the USA for this indication.

As of today, gefitinib, erlotinib, afatinib, dacomitinib, and osimertinib are all FDA-approved first-line treatment of patients with metastatic NSCLC whose tumors have EGFR exon 19 deletions or exon 21 L858R mutations (Table 1). Afatinib was also active in non-small cell lung cancer tumors that harbored certain types of uncommon EGFR mutations, especially Gly719Xaa, Leu861Gln, and Ser768Ile, but less active in other mutations types.

In a phase 3 clinical trial, osimertinib significantly prolonged progression-free survival to 18.9 months as compared to 10.2 months for patients with untreated EGFR-mutated advanced NSCLC received gefitinib or erlotinib. The response rate, 76-80%, is similar for all three agents. However, the duration of response is much longer with osimertinib (17.2 months) than with gefitinib/erlotinib (8.5 months). Additionally, grade 3 or 4 adverse events are less frequently reported with osimertinib when compared with gefitinib/erlotinib. Recently, results from FLAURA study revealed that the median overall survival was 38.6 months in patients who received osimertinib in comparison to 31.8 months in patients who received gefitinib/erlotinib. These results indicate osimertinib demonstrates better efficacy and less side effects, as well as CNS penetration, making it the new standard first-line treatment for mutated EGFR advanced NSCLC patients [9].

Nazartinib (EGF816), BPI-7711, lazertinib (YH25448), and HS-10296 are all third-generation oral EGFR TKIs selective for activating (L858R, ex19del) and resistance (T790M) mutants.

In a phase II trial, 40 treatment-naïve EGFR mutant advanced NSCLC patients received 150 mg of oral nazartinib daily; 16 of the 40 patients (40%) had brain metastases. The objective response rate (ORR) was 67% (1 CR/15 PRs); disease control rate was 96%. Duration of response and progression-free survival (PFS) data were still immature at the data cut-off. The most common adverse events were maculopapular rash, diarrhea, and stomatitis [12].

A phase I trial examining the benefit of treatment with BPI-7711, that included 85 patients with advanced or recurrent EGFRm+/T790M+ NSCLC who had progressed after 1st/2nd generation of EGFR TKIs were enrolled into 5 dose escalation cohorts (30/60/120/180/240 mg). No DLT was observed. The most common adverse events were white blood cell count decrease, neutrophil count decrease, upper respiratory infection, vomiting, and diarrhea. Serious adverse events were reported in 4% of patients. Fifty-five patients were available for efficacy evaluation. The ORR was 54.5% (30/55) including 1.8% CR and 52.7% PR. The disease control rate was 96.4% [13].

A total of 127 EGFRm and T790M resistance advanced NSCL patients who progressed after treatment with standard EGFR TKIs were enrolled into a phase I/II trial of YH25448. The treatment dose of YH25448 ranged from 20 to 320 mg. No DLT was observed. The most common adverse events were pruritus, decreased appetite, rash, and constipation. Grade 3 or higher adverse events were observed in 3% of patients. Median duration of treatment was 9.7 months. In patients evaluated for efficacy, the ORR was 60%. The ORR was 64% in T790M-positive patients compared with 37% of patients in T790M negative. The ORR was 50% in patients with brain metastasis (*n* = 14). The median PFS was 8.1 months in all patients, 9.5 months in T790M-positive patients, and 5.4 months for T790M negative patients. In patients who received higher than 120 mg doses, the ORR was 65% and the PFS was 12.2 months [14].

In a phase I trial examining the treatment benefits of HS-10296, a total 117 patients with EGFRm and T790M resistance advanced NSCL patients who progressed after treatment with standard EGFR TKIs were enrolled. The treatment dose of HS-10296 ranged from 55 to 260 mg. The MTD has not been reached and the most common adverse events were rash, pyrexia, upper respiratory tract infection, constipation, and diarrhea. Efficacy was evaluated in 82 patients. The ORR was 52.5% and the disease control rate (DCR) was 91.5%. The DCR in patients receiving 110 mg improved to 97.2%. Thus, the recommended phase II dose was 110 mg [15].

### EGFR TKIs targeting exon 20

Patients with EGFR/HER2 exon 20 mutations account for about 10% of all EGFR-mutated NSCLC. The presence of these mutations usually confers primary resistance to TKIs.

Recently, two new targeted agents showed activity in this subtype of NSCLC, TAK-788, and poziotinib. TAK-788 is an investigational TKI that inhibits the EGFR and HER2 receptors. In a phase I/II clinical trial, 101 patients received TAK-788 treatment. The treatment dose of TAK-788 ranged from 5 to 180 mg. The phase II recommended dose was 160 mg. Efficacy was evaluable in 24 patients with EGFR exon 20 insertions. Twenty-three had decreased target lesion measurements with median percent change of 32.6%. The ORR was 54% in patients that received 160 mg. Adverse event profile was similar with other EGFR TKIs [16, 17].

A phase II clinical trial with poziotinib enrolled 50 patients in an *EGFR* cohort; 40 patients were evaluable for response. The overall response rate is 58% and the DCT was 90%. Eight out of 13 responders (62%) were previously treated with a TKI. Thirteen patients enrolled to the HER2 cohort and 12 patients were evaluated for response. The ORR was 50% and the DCR is 83% (World Lung 2018 Abstract OA02.06).

### Resistance after EGFR TKIs treatment

Most of the patients who received EGFR TKIs with initial response will eventually develop disease progression. For patients who had disease progression after gefitinib, erlotinib, or afatinib, about half of the patients develop resistance related to EGFR T790M. Patient usually will be given osimertinib to overcome EGFR T790 M resistance.

For patients who had disease progression after osimertinib, there is EGFR-dependent and EGFR-independent resistance. In EGFR-dependent resistance, about half of the patient lost EGFR-T790M mutation.

The second common mechanism of resistance is acquired amplification of MET which could occur in about 16% of patients who had disease progression after gefitinib or erlotinib, and it could happen up to 30% of patients who treated after osimertinib. The other resistance mechanisms to EGFR TKIs therapy include HER2 amplification, RAS/MAPK/PI3K pathway activation, cell cycle gene alteration, and transformation of into small cell lung cancer [18–20].

For patients who have progressed after osimertinib, there is no FDA-approved targeted therapy. The current standard is to give chemotherapy or chemotherapy plus immunotherapy such as IMpower 150 regimen.

For patients who had progressed after osimertinib with MET-driven acquired resistance, a phase Ib SAVANNAH study showed an efficacy of osimertinib plus MET inhibitor with ORR 64–66%. However, there are about 38–57% of patients experienced grade 3 or more adverse events. Some patients experience anaphylactic reaction related to savolitinib. Currently, phase II SAVANNAH study is on hold due to safety concerns [21].

### ALK fusion/rearrangement inhibitors

The EML4 and ALK genes are within the short arm of chromosome 2; inversion of these 2 genes resulted in the novel fusion oncogene EML4-ALK. It is found approximately in 2–7% of advanced NSCLC patients, typically in younger and never smokers [22, 23].

**Table 2:** FDA-approved ALK TKIs

Drug	Month/year	Indication	Comments
Crizotinib	August/2011	Metastatic ALK+ NSCLC	First generation
Ceritinib	April/2014	Metastatic ALK+ NSCLC	Second generation
Alectinib	December/2015	Metastatic ALK+ NSCLC who have progressed on or are intolerant to crizotinib	Second generation
	November/2017	Metastatic ALK+ NSCLC	
Brigatinib	April/2017	Metastatic ALK+ NSCLC who have progressed on or are intolerant to crizotinib	Second generation
Lorlatinib	November/2018	Metastatic ALK+ NSCLC who has progressed on alectinib or ceritinib, or crizotinib and at least one other ALK inhibitor	Third generation

Crizotinib is the first approved targeted therapy for ALK-positive advanced NSCLC. It has shown prolonged PFS when compared with chemotherapy. The median PFS with Crizotinib is 10.9 months versus 7.0 months with chemotherapy [24]. However, resistance invariably develops and crizotinib has poor CNS penetration as well. Newer generations of ALK inhibitors are more potent than crizotinib and can overcome the resistance (Table 2). ALK inhibitors, such as alectinib, brigatinib, and lorlatinib also showed intracranial efficacy.

Currently, the preferred first-line treatment is alectinib based on the ALEX trial. A total of 303 untreated ALK-positive advanced NSCLC patients were randomized to receive either alectinib or crizotinib. The 12-month event-free survival rate was 68.4% with alectinib compared to 48.7% with crizotinib. The median PFS was 34.8 months with alectinib and 10.9 months with crizotinib. The PFS hazard ratio was 0.43. The median PFS with baseline CNS metastasis was 27.7 months with alectinib in comparison to 7.4 months with crizotinib. Alectinib was also better tolerated than crizotinib [25, 26].

Brigatinib is another next-generation ALK inhibitor that showed improved PFS over crizotinib, with an estimated 12-month PFS of 67% compared to crizotinib 12 month PFS of 43%. The hazard ratio with brigatinib was 0.49. The confirmed ORR was 71% with brigatinib compare to 60% with crizotinib. The intracranial response rate was 78% over 29%, respectively. No new safety concerns were reported [27].

Finally, ceritinib is more potent than crizotinib and showed efficacy in patients who have progressed on crizotinib. However, ceritinib was not compared head-to-head with crizotinib in the first-line setting [28].

Lorlatinib is a third generation inhibitor of ALK and ROS1. In a phase II trial, a subgroup of 198 advanced ALK-positive NSCLC patients who had progressed on one or more ALK inhibitors were enrolled. The ORR was 47%. Objective intracranial response was 63%. The most common adverse effects were hypercholesterolemia, hypertriglyceridemia, edema, and peripheral neuropathy. Serious adverse events occurred in 7% of patients [29].

Ensartinib (X-396) is a potent ALK TKI and has also shown inhibitory activity against MET, BAL, Axl, EPHA2, LTK, ROS1, and SLK. In a phase I/II study, 97 patients with advanced ALK-positive NSCLC were enrolled and given ensartinib 225 mg once daily. Common toxicities were rash, nausea, pruritus, vomiting, and fatigue. For evaluable patients, the response rate was 60% and the median PFS was 9.2 months. For ALK TKI naïve patients, RR was 80% and median PFS was 26.2 months. Patients with brain metastases were observed with intracranial RR of 64% [30]. A phase III clinical trial to compare ensartinib to crizotinib in ALK-positive NSCLC patients is currently open and enrolling patients [31].

### ROS1/NRTK inhibitor

#### ROS1

ROS1 rearrangement accounts for about 1–2% in NSCLC and typically occurs in younger patients who are never smokers. The ROS1 locus is located on chromosome 6 and encodes for a tyrosine kinase receptor.

Crizotinib was approved for metastatic ROS1 positive NSCLC in March 2016 (Table 3). In an ongoing phase I study, crizotinib showed anti-tumor activity in patients with ROS1 positive advanced NSCLC with the ORR was 72% and median duration of response was 17.6 month. Median PFS was 19.3 months. Median OS was 51.4 months [32].

Ceritinib and lorlatinib were not approved in metastatic ROS1 +NSCLC yet but both showed anti-tumor activity in this patient population. In a phase II trial conducted in Korea, 32 patients with ROS1 positive advanced NSCLC were treated with ceritinib. Two patients had previously received crizotinib while 30 patients were crizotinib naïve. Twenty-eight patients were evaluated for efficacy. The ORR was 62% and the DCT was 81%. The median PFS was 9.3 months for all patients and 19.3 months for crizotinib naïve patients. The median OS was 24 months [33].

In a phase II trial, lorlatinib was given to 47 patients with ROS1+ advanced NSCLC. Thirty-four patients were previously treated with crizotinib and 13 patients were crizotinib naïve. Twenty-five patients had baseline CNS metastases. The ORR was 36.2% in all patients and 61.5% in crizotinib naïve patients. The intracranial ORR was 56% in all patients. The median PFS was 9.9 months in all patients and 21 months in crizotinib naïve patients [34].

#### NTRK

NTRK gene fusions involve either NTRK1, NTRK2, or NTRK3 and occurs across tumor types. These fusions account for about 1% in NSCLC. The first approved oral TKI for NRTK was larotrectinib. Larotrectinib is approved for advanced or metastatic solid tumors that harbor the NTRK fusion genes with conditions found in Table 3. A trial that combines 3 phase I/II trials reported integrative efficacy and safety for larotrectinib in NTRK fusion-positive cancers in adults and children. The ORR was 75%. The median duration of response and PFS were not reached. Most treatment-emergent adverse events (TEAEs) were grade 1, and grade 3/4 TEAEs were reported in less than 5% of patients [35].

**Table 3:** FDA-approved ROS1 TKI and NTRK TKI

Drug	Month/year	Indication	Comments
Crizotinib	March/2016	Metastatic ROS1 +NSCLC	
Larotrectinib	November/2018	Advanced or metastatic NTRK gene fusion Solid tumors, no acquired resistance mutation, and have no satisfactory alternative treatments available	Adult and pediatric patients
Entrectinib	August/2019	Adult metastatic ROS1 +NSCLC, adult and pediatric patients 12 years of age and older with advanced or metastatic NTRK gene fusion solid tumors, no acquired resistance mutation, have progressed following treatment or have no satisfactory alternative therapy, available	

Entrectinib is an oral TKI for ROS1, ALK, and NTRK gene fusions. Entrectinib was first approved in Japan in June 2019 for the treatment of adult and pediatric advanced or recurrent NSCLC with NTRK fusions. Recently, the FDA-approved entrectinib for similar indication as well as for ROS1-positive advanced NSCLC (Table 3). In an integrated analysis of 3 phase I/II trials, entrectinib demonstrated 50% ORR in patients with NTRK fusion-positive solid tumors and 70% in ROS1-positive NSCLC patients. The intracranial ORR was 55% in both ROS1-positive NSCLC patients and NTRK fusion-positive solid tumor patients. The median duration of response ranged from 12.6 to 24.6 months [36, 37].

DS-6051b is a TKI with high affinity for ROS1 and NTRK kinases/In a phase I/Ib trial, it was found to be well tolerated and had a signal of inhibitory activity. Six patients were evaluated for efficacy, Two had PR and 2 had stable disease (SD) [38].

Repotrectinib (TPX-0005) is a potent next-generation ALK, ROS1, or NTRK1-3 fusion TKI. It is greater than 90-fold more potent than crizotinib. Repotrectinib demonstrates overall and intracranial anti-tumor activity in ROS1 positive advanced NSCLC patients.

In a phase I trial, 65 patients who had either ALK+, ROS1+, or NTRK+ advanced solid tumors were included, 23 patients had baseline CNS disease. The most common TEAEs were dysgeusia, dizziness, paresthesia, and nausea. DLTs occurred in 2 NSCLC patients at 240 mg daily and 160 mg daily dose. MTD has not been reached. Confirmed partial response was observed in 8 patients with ROS1+/NTRK+. For ALK + patients, 4/16 had stable disease [39]. In the updated report, 75 patients were enrolled. Twenty-eight patients were ROS1+. The ORR was 90% in 10 TKI-naïve patients and 28% in 18 TKI pre-treated patients. The response rate was 44% in 9 TKI pre-treated patients who received a repotrectinib dose of 160 mg or above. The intracranial ORR was 100% in 3 TKI naïve patients and 50% in 4 TKI pre-treated patients [40].

### BRAF V600E mutations

An activating BRAF mutation occurs in 1–3% of NSCLC patients with a related history of smoking. The combination of dabrafenib and trametinib was approved for the treatment of metastatic NSCLC harboring BRAF V600 E mutations based on an open-label trial. A total of 93 patients were enrolled in the trial. Fifty-seven of the 93 patients received previous systemic treatment and 36 patients were treatment naïve. All patients received dabrafenib 150 mg oral twice daily and trametinib 2 mg orally once a day. The ORR was 63% in pretreated patients and a response duration of ≥ 6 months was observed in 64% of responders. In treatment-naïve patients, the ORR was 61% and the response duration of ≥ 6 months was observed in 59% of responders (Table 4) [41, 42].

**Table 4:** FDA-approved targeted therapy for BRAF mutations

Drug	Month/year	Indication	Comments
Dabrafenib+trametinib	June/2017	Metastatic BRAF V600E+ NSCLC	

### MET inhibitors

MET is tyrosine kinase receptor binding with hepatocyte growth factor (HGF). MET gene amplification and exon-14-skipping mutations are characteristic abnormalities causing increased MET signaling activation. Isolated MET exon 14 mutation is found in 3% of NSCLC; however, it is an acquired EGFR TKI resistance pathway in 15–20% of EGFR mutation-positive NSCLC cases [43]. Crizotinib and cabozantinib have been shown to have some activity against MET [44, 45].

Capmatinib is a highly selective MET inhibitor. In a phase II trial, Capmatinib was given to advanced NSCLC patients with MET exon-14-skipping mutations or MET amplifications. Ninety-seven MET exon-14 NSCLC patients were evaluable for efficacy. The ORR was 39.1% in pretreated patients and 71.4% in treatment-naïve patients. The most common adverse events were peripheral edema, nausea, and vomiting [46].

Tepotinib is another highly selective MET inhibitor. In a phase II trial, tepotinib single agent was given as first-line to advanced NSCLC patients with MET exon 14 mutations identified through liquid biopsy or tumor biopsy. Eighty-five patients were enrolled and 76 patients were evaluable for efficacy. The ORR was 51.4% in liquid biopsy patients and 41.5% tumor biopsy patients. The most common TEAEs were peripheral edema, diarrhea, nausea, and asthenia. TEAEs lead to discontinuation in 2 patients, one due to interstitial lung disease and another one due to nausea and diarrhea [47].

### RET fusion/rearrangement inhibitors

RET gene encodes a receptor tyrosine kinase (RTK) belonging to the *RET* family of RTKs. RET rearrangements are found in 1–2% lung adenocarcinoma and are mutually exclusive with mutations involving EGFR, ALK, ROS1, BRAF, or KRAS.

The most common fusion partner for RET rearrangements in patients with NSCLC is KIF5B.

Multi-targeted TKIs (MKIs) such as cabozantinib, vandetanib, lenvatinib, sunitinib, and target RET fusion-driven NSCLCs have been tested. The most studied RET inhibitors are cabozantinib and vandetanib, which both showed a response rate between 20 to 50% in patients with RET-rearranged NSCLC [48–50]. Lenvatinib and sunitinib have been used in fewer patients with reported response rate at 16–22% [51, 52]. These agents are approved for other indications but are not designed to selectively target RET. Given the low response rate to Multi-targeted TKIs in RET-rearranged NSCLC, the next-generation RET-selective inhibitors, RXDX-105, BLU-667, and LOXO-292, are being tested.

RXDX-105 is a VEGFR-sparing potent RET inhibitor. In a phase Ib study, 21 untreated RET fusion-positive NSCLC patients received RXDX-105. Of these, eight patients harbored non-KIF5B-RET fusions and six out eight patients (75%) achieved response. None of the 13 patients with KIF5B-RET fusions had a RECIST response [53].

BLU-667 demonstrated ≥ 10-fold increase in potency over the FDA-approved MKIs, against oncogenic RET rearrangements. In a phase I study, seven out of 14 (50%) RET fusion NSCLC patients who received BLU-667 achieved objective response [54].

In an updated report, 79 advanced RET fusion+ NSCLC patients received BLU-667. The ORR was 56% in 57 evaluable patients. The DCR was 91%. Six patients had response duration ≥ 6 months. The Most commonly reported TEAEs are increased AST, hypertension, increased ALT, constipation, fatigue, and decreased neutrophils [55].

LOXO-292 is another RET-selective TKI. A phase I basket trial of LOXO-292 in RET-driven cancers was presented at the American Society of Clinical Oncology Annual Meeting in 2018. Fifty-seven patients were treated with LOXO-292. Twenty-seven of the 57 were patients with NSCLC. The ORR in evaluable RET fusion-positive patients was 69% (22/32) and 65% (17/26) in NSCLC. The median duration of response has not been achieved but is estimated to be greater than 6 months [56].

RXDX-105, BLU- 667, and LOXO-292 also demonstrated some central nervous system penetration in these early-phase trials.

### HER2

Human epidermal growth factor receptor 2 (HER2; ERBB2) is a member of the tyrosine kinase receptor family, which also includes HER1, HER3, and HER4.

HER2-targeted therapy has showed improved survival in breast cancer and gastroesophageal cancers. In the Lung Cancer Mutation Consortium (LCMC), HER2 mutations account for about 3% of patients with lung adenocarcinoma [57].

In a single-arm phase II study, trastuzumab-paclitaxel showed anti-tumor activity with an ORR of 46% (11/24) of EGFR TKI pretreated patients with an activating EGFR mutation and HER2 mutation.

Recently, a phase II basket trial evaluated ado-trastuzumab emtansine for patients with HER2-mutant lung adenocarcinomas. Eight out of 18 patients (44%) responded. The median PFS was 5 months [58].

Trastuzumab deruxtecan (DS-8201a) is a HER2-targeting antibody-drug conjugate with a topoisomerase I inhibitor. In December 2019, trastuzumab deruxtecan was approved by FDA for patients with unresectable or metastatic HER-2 breast cancer who have received two or more prior anti-HER2-based treatments. In a large phase 1 trial, it also showed efficacy in HER-2 expression solid tumors other than breast cancer or gastric cancer. ORR was 36.4% (8/22) and DCR was 81.8% (18/22). Median duration of response and median PFS were not reached. Common TEAEs were nausea, vomiting, and decreased appetite [59, 60].

In an international multicenter retrospective study, 27 patients with stage IV or recurrent HER2-mutated NSCLC were treated with afatinib. The ORR was 13% (3/23 evaluable patients) and the median overall survival from the date of diagnosis of metastatic or recurrent disease was 23 months [61].

In a phase II trial of dacomitinib in patients with HER2-mutant or amplified tumors, partial response was observed in 3 of 26 patients with HER2 exon 20 mutations and no response was observed in 4 patients with HER2 amplifications. The median overall survival was 9 months from start of dacomitinib for patients with HER2 mutations.

XMT-1522, a novel HER2-antibiody-drug conjugate, is an auristatin-derivative conjugated to a novel anti-HER2 (Dolaflexin). A phase 1 trial showed that the drug conjugate is well tolerated up to 21.3 mg/m2, and showed early signs of anti-tumor activity. The DCR was 5/6 (83%) for patients dosed at 16 or 21.3 mg/m2 with 1 PR and 4 SD [62].

Pyrotinib is an oral pan-TKI that inhibits HER1, HER2, and HER4. In a phase II trial, 60 patients with advanced NSCLC with HER2 exon 20 mutations who had disease progression after platinum-based chemotherapy were enrolled to receive pyrotinib 400 mg once daily. The ORR was 31.7% and the median duration of response was 7.0 months. The median PFS was 6.8 months. Grade 3 TEAEs occurred in about 26.7% patients [63, 64].

As previously mentioned, 13 patients were enrolled to HER2 cohort of poziotinib phase II clinical trial and 12 patients were evaluated for response. The ORR is 50% and the DCR is 83%.

### KRAS G12C inhibitor

KRAS mutations represent one of the most common oncologic driver mutations in advanced NSCLC. The KRAS G12C mutation accounts for about 14% of lung adenocarcinoma. Patients with KRAS + NSCLC have a shorter median survival. Until recently, no effective treatment against KRAS has been identified.

AMG510 is a small promising molecule that selectively inhibits KRAS G12C by locking it in an inactive GDP-bound state. A phase 1 study evaluated AMG 510 in adult patients with locally advanced or metastatic KRAS G12C mutation solid tumors. Thirteen NSCLC patients were enrolled to the study. These patients have a median of 3 lines of previous treatments. Ten patients were evaluated for efficacy. Five had a PR with ORR about 50%, 4 had SD and 1 had PD. Six NSCLC patients reported 10 TEAEs (6 grade 1, 2 grade 2, and 2 grade 3). The grade 3 TEAEs were anemia and diarrhea. The most common AEs were decreased appetite and diarrhea [65, 66].

MRTX849 is another KRAS G12C inhibitor showing activity in treating advanced solid malignancies with KRAS G12c mutations. In a phase I/II study, 17 patients received MRtx849 with dose ranged from 150 mg to 1200 mg daily. Of 10 NSCLC patients treated, 6 patients were evaluable and 3 achieved PR with ORR about 50%. Common TEAEs were diarrhea, nausea, vomiting, elevated liver enzymes, increased creatinine, and decreased appetite. Grade 3 toxicities were fatigue, decreased appetite, and dyspnea [67].

Single-agent and combination studies are ongoing for AMG510 and MRTX849.

### ATR inhibitor

ATR is a critical component in DNA damage response and inhibition of ATR signaling may sensitize tumors to DNA-damaging chemotherapy. M6620 is a selective inhibitor of ATR. M6620 monotherapy is well tolerated but showed limited anti-tumor activity. Thirty-three patients with advanced NSCLC who had received up to 2 lines of prior therapies were enrolled in a phase I trial of M6620 plus gemcitabine. Grade 3 or higher TEAEs occurred in 19/33 (57.6%) patients and the most commonly reported TEAEs were fatigue, neutropenia, anemia, and thrombocytopenia. Efficacy was evaluable in 24 patients. The ORR was 12.5% and 18 patients had SD [68].

### AXL kinase inhibitor

AXL overexpression may induce epithelial to mesenchymal transition, tumor angiogenesis, resistance to chemotherapeutic and targeted agents, and decreased anti-tumor immune response. Inhibition of AXL by TP-0903 may potentially reduce cancer cell metastasis, reverse resistance to immunotherapy and targeted therapy, and activate the anti-cancer immune response.

A phase Ia/Ib study to evaluate safety and PK/PD of TP-0903, a potent inhibitor of AXL kinase in advanced solid tumors is ongoing. Results are not yet available [69].

Bemcentinib is an oral highly selective inhibitor of the AXL tyrosine kinase. Thirty-eight advanced NSCLC patients who had progressed on one line of platinum-based chemotherapy or first-line of targeted therapy of EGFR inhibitor or ALK inhibitor were enrolled in a phase II trial of bemcentinib in combination with pembrolizumab. The most common TEAEs were transaminase increases, diarrhea, and asthenia. Efficacy was evaluable in 29 patients. The ORR was 24%. For AXL-positive patients, the ORR was 40%. The median PFS was 4.0 months and 5.9 months for AXL-positive patients [70].

In a phase I/II clinical trial, bemcentinib and docetaxel combination was given to patients with previously treated non-squamous NSCLC. Eleven patients were treated. Two DLTs were observed in a dose of 75 mg/m2 docetaxel and 100 mg daily of bemcentinib; thus, the ongoing recruitment was continued with a dose of 60 mg/m2 docetaxel and 100 mg daily of bemcentinib. Efficacy was evaluable in 7 patients, 2 achieved PR and 2 had SD [71].

### VEGF/VEGEFR

MP0250 is a tri-specific DARP in compound neutralizing VEGF-A and HGF as well as binding albumin and increasing plasma half-life. A phase I trial of MP0250 resulted in a MTD of 8 mg/kg and was well tolerated. Two patients had PR, which indicated signs of anti-tumor activity [72].

**Fig. 1:**
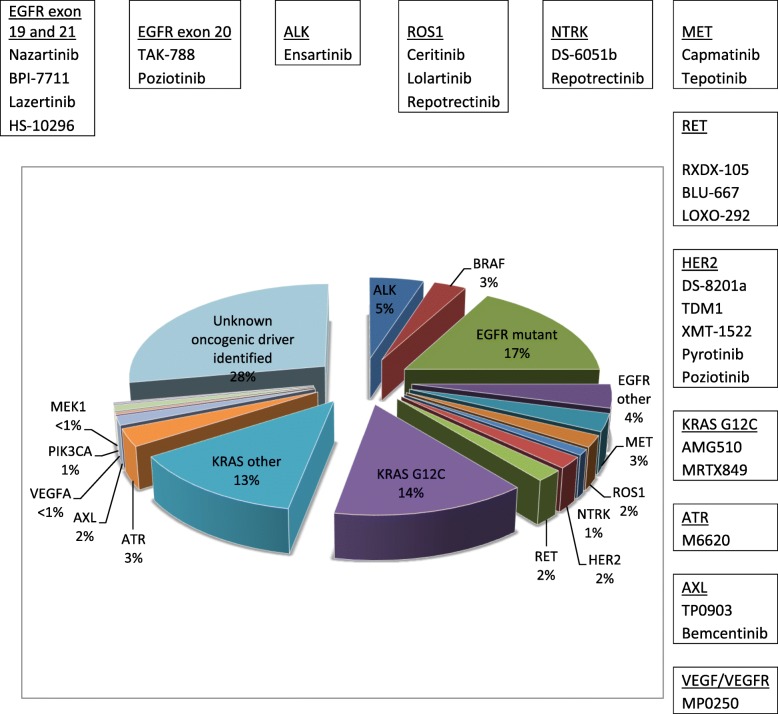
Mutation frequency of lung adenocarcinoma and emerging drugs targeted these mutations. Frequency data is a combination from the AACR GENIE data and Lung Cancer Mutation Consortium [73, 74]

Figure 1 briefly summarized the emerging targeted therapy discussed above.

## Antibody-drug conjugates

JNJ-372 is an EGFR-cMet bispecific antibody that showed activity in EGFRm NSCLC. In a phase 1 study, advanced EGFRm NSCLC patients, including patients who have progressed on 3rd generation EGFR TKI and EGFR Exon 20 disease, were enrolled. Of 116 treated NSCLC patients, common adverse events were rash, infusion-related reaction, paronychia, and constipation. Efficacy was evaluated in 88 patients. The ORR was 28%. PR was observed in 10/47 patients with prior 3rd-generation EGFR TKI. PR was observed in 6/20 patients with EXON 20 [75].

DS-1062a is an antibody-drug conjugate that is comprised of trophoblast cell-surface antigen 2 (TROP2)-targeting antibody attached to topoisomerase I inhibitor payload by a tetrapeptide-based linker. Overexpression of TROP2 may be related to poor survival in solid tumor patients. A phase I study of DS-1062a in patients with advanced solid tumors is ongoing. Twenty-two patients with advanced NSCLC received treatment of DS-1062a at doses of 0.27–2.0 mg/kg. Median of 2 cycles was initiated. Grade 1 TEAEs were experienced in 81.8% of patients. Fatigue was the most commonly reported TEAE and was the only grade 3 TEAE. Efficacy was evaluated in 18 patients, 1 achieved PR and 8 achieved SD [76].

EnaV is anti-AXL humanized IgG1 conjugated with monomethyl auristatin E. In a phase I/II clinical trial, heavily pretreated solid tumor patients with relapsed or refractory disease were enrolled to received monotherapy of EnaV. The recommended phase II dose was 2.2 mg/kg once every 3 weeks. Twenty-six advanced NSCLC patients who are EGFR and ALK wide type and failed ≤ 4 lines of systemic therapies including platinum-based chemotherapy and PD-1/PD-L1 inhibitor were enrolled. Nine out 12 evaluable fresh biopsies were AXL-positive. The ORR is 19% and DCR is 50%. The most common grade 3 or 4 TEAEs were gastrointestinal disorders, nausea, vomiting, and diarrhea [77].

An antibody-drug conjugate PF-06647020 (PF-7020) targeting PTK7 (protein tyrosine kinase 7) was evaluated in a phase I trial, in advanced solid malignancies; the antibody-drug conjugate results indicate a manageable safety profile and promising activity. Four of 23 NSCLC patients had PR; 10/23 had SD [78].

Anetumab ravtansine is a human anti-mesothelin IgG1 antibody conjugated to the maytansinoid tubulin inhibitor DM4. A phase 1b study of anetumab ravtansine in patients with mesothelin-expressing advanced or recurrent malignancies is ongoing and results are not yet available [79].

BA3011 is an anti-AXL humanized monoclonal antibody conjugated to monomethyl auristatin E using a cleavable linker (CAB-AXL-ADC). Preclinical data showed BA3011 has anti-tumor activity in NSCLC, pancreatic, castration-resistant prostate cancer (CRPC), and other tumor models. A phase I study of BA3011 in patients with advanced solid tumors is ongoing and results are not yet available [80].

BT1718 is a bicycle drug conjugate comprising a bicyclic peptide that binds membrane type I matrix metalloproteinase (MT1-MMP) and is linked to maytansinoid tubulin inhibitor DM1 by a cleavable disulfide linker. MT1-MMP overexpression was seen in NSCLC. A phase I/IIa trial of BT1718 in patients with advanced solid tumors is ongoing and results not yet available [81].

High expression of HER3/ERBB3 in NSCLC may be correlated with poorer outcomes. U3-1402 is a HER3-targeting antibody-drug conjugate (ADC), composed of patritumab monocloncal antibody directed against HER3 linked to the topoisomerase I inhibitor DX8951. A current phase I clinical trial of U3-1402 in metastatic or unresectable adenocarcinoma NSCLC subjects harboring EGFR-activating mutation who (a) are T790M mutation-negative after disease progression during treatment with erlotinib, gefitinib, or afatinib or (b) develop disease progression while on osimertinib. Results are not yet available [82].

## Immunotherapy

Cancer immunotherapy therapy works by empowering our own immune system to fight against cancer. It is a rapidly advancing and an important force in the fight against cancer along with surgery, radiation, chemotherapy, targeted therapy, and endocrine therapy.

Immunotherapy can be activated through vaccine therapies to stimulate the immune system to attack cancer cells (active immunotherapy) or through checkpoint inhibitors to remove the immune blockage (passive immunotherapy).

Using our own immune system to treat cancer was first noted in the nineteenth century by Dr. William Coley. Dr. Coley observed some cancer patients went into spontaneous remission after erysipelas infection. He began injecting mixtures of live and inactivated bacteria (Coley’s mixed bacterial toxin) into patients’ tumors in 1891. Coley reported a significant number of patients with cancer achieved response and cure. Today Dr. Coley is known as the Father of immunotherapy. His method gained acceptance but then gradually disappeared with the development of radiation and chemotherapy as well as the risk associated with infecting cancer patients with bacteria [83].

Another early cancer immunotherapy, Bacillus Calmette-Guérin (BCG) was used in the treatment of superficial bladder cancer. First reported by Dr. Old, BCG was used as a treatment in a mice model in 1959. Dr. Old is now known as Father of Modern Tumor Immunology. Thomas and Burnet proposed the theory of immunosurveillance. In 1957, they hypothesized that lymphocytes act as sentinels in recognizing and eliminating continuously arising, nascent transformed cells [84, 85].

The first approved immunotherapy in metastatic NSCLC was nivolumab. Nivolumab is a fully human IgG4 PD-1 immune-checkpoint-inhibitor antibody. It binds to PD-1 receptor this block tumor PD-L1 to bind with T cell PD-1 receptor thus restores anti-tumor immunity. In CheckMate 017, a phase III clinical trial, patients with metastatic squamous cell NSCLC who had disease progression after platinum-based chemotherapy were randomized to receive nivolumab or docetaxel. The median OS was significantly improved for nivolumab (9.2 vs 6.0 months), as well as response rate (20% vs 9%) and median PFS (3.5 vs 2.8 months) [86].

Table 5 shows FDA-approved immunotherapies. Both nivolumab and pembrolizumab are PD-1 immune-checkpoint-inhibitor antibodies. Atezolizumab is a fully humanized IgG1 antibody against PD-L1.

**Table 5:** FDA-approved immunotherapy in advanced/metastatic NSCLC

Drug	Class	Month/year	Indication(s)
Nivolumab (OPDIVO)	Anti-PD-1	March/2015	Metastatic squamous NSCLC with progression on or after platinum-based chemotherapy.
		October/2015	Metastatic NSCLC in patients with progression on or after platinum-based chemotherapy. Patients with EGFR or ALK genomic tumor aberrations should have disease progression on FDA-approved therapy for these aberrations prior to receiving OPDIVO.
Pembrolizumab (KEYTRUDA)	Anti-PD1	October/2015	Metastatic NSCLC whose tumors express PD-L1 (TPS ≥ 1%) with progression or after platinum-containing chemotherapy. Patients with EGFR or ALK genomic tumor aberrations should have disease progression on FDA-approved therapy for these aberrations prior to receiving KEYTRUDA.
		October/2016	NSCLC whose tumors have high PD-L1 expression (TPS ≥ 50%), with no EGFR or ALK genomic tumor aberrations, and no prior systemic chemotherapy treatment for metastatic NSCLC.
		May/2017	In combination with pemetrexed and carboplatin, as first-line treatment of patients with metastatic non-squamous NSCLC and with no EGFR or ALK genomic tumor aberrations.
		October/2018	In combination with carboplatin and either paclitaxel or nabpaclitaxel, as first-line treatment of patients with metastatic squamous NSCLC.
		April/2019	Single agent for the first-line treatment of patients with stage III NSCLC, who are not candidates for surgical resection or definitive chemoradiation, or metastatic NSCLC, and whose tumors express PD-L1 [tumor proportion score (TPS) ≥ 1%] as determined by an FDA-approved test, with no EGFR or ALK genomic tumor aberrations.
Atezolizumab (TECENTRIQ)	Anti-PD-L1	October/2016	Metastatic NSCLC who have disease progression during or following platinum-containing chemotherapy. Patients with EGFR or ALK genomic tumor aberrations should have disease progression on FDA-approved therapy for these aberrations prior to receiving TECENTRIQ.
		December/2018	In combination with bevacizumab, paclitaxel, and carboplatin, for the first-line treatment of adult patients with metastatic non-squamous NSCLC with no EGFR or ALK genomic tumor aberrations.
		December/2019	In combination with paclitaxel protein-bound and carboplatin for the first-line treatment of adult patients with metastatic non-squamous non-small cell lung cancer (NSCLC) with no EGFR or ALK genomic tumor aberrations.

*TPS* tumor proportion score

Pembrolizumab has been FDA-approved in metastatic NSCLC second-line setting if PD-L1 tumor proportion score (TPS) > 1%. It was also approved in first-line metastatic NSCLC with PD-L1 TPS > 50% after a phase 3 trial showed significantly longer progression-free survival and overall survival and less side effects when compared with platinum-based chemotherapy. In the KEYNOTE-042 study, pembrolizumab monotherapy was compared with platinum-based chemotherapy in first-line therapy for advanced/metastatic NSCLC with PD-L1 TPS ≥ 1%. Pembrolizumab significantly improved OS in patients with TPS ≥ 50% (20 months vs 12.2 months, HR 0.69), TPS ≥ 20% (17.7 months vs 13 months, HR 0.77), and TPS ≥ 1% (16.7 months vs 12.1 months, HR 0.81) [87].

More recently, pembrolizumab in combination with chemotherapy showed an improved OS irrespective of PD-L1 status when compared to chemotherapy. The benefits can be seen in both non-squamous and squamous histology type. In non-squamous, the hazard ratio for death is 0.49 and the 12-month overall survival is 69.2%. In squamous, the hazard ratio for death is 0.64 with a median overall survival of 15.9 months in pembrolizumab-chemotherapy combination group compare to chemotherapy group.

Recently, atezolizumab in combination with bevacizumab, paclitaxel, and carboplatin was approved for the first-line treatment of adult patients with metastatic non-squamous NSCLC with no EGFR or ALK genomic tumor aberrations. In a phase 3 randomized trial, atezolizumab (A) in combination with bevacizumab, paclitaxel, and carboplatin (BCP) showed longer median PFS (8.3 months) than the BCP group (6.8 months). The Hazard ratio for disease progression or death is 0.62 with 95% confidence interval 0.52 to 0.74 [88].

### Anti-PD1/PD-L1 and anti-CTLA-4

In a phase III trial, first-line treatment with nivolumab plus ipilimumab in advanced NSCLC showed improved overall survival compare with patients treated with chemotherapy, regardless of PD-L1 expression. For patients treated with nivolumab+ipilimumab, median OS was 17.1 months (PD-L1 expression > 1%) and 17.2 months (PD-L1 < 1) compare to 14.9 months (PD-L1 > 1%) and 12.2 months (PD-L1 < 1%) in patients treated with chemotherapy [89].

A phase 1A/1B study (NCT02407990) showed single-agent tislelizumab (anti-PD-1) was well tolerated and showed evidence of anti-tumor activity in patients with solid tumors, including NSCLC. In a phase II trial, tislelizumab in combination with chemotherapy was evaluated as a first-line treatment in Chinese patients with advanced NSCLC. Fifty-four patients were enrolled in the study. The ORR was 67% (36/54). Grade 3/4 AEs occurred in > 15% patients. The most commonly reported AEs were decreased neutrophil counts and anemia [90].

Cemiplimab is a human monoclonal anti-PD-1 and was approved for advanced and metastatic cutaneous squamous cell carcinoma not suitable for curative surgery or radiation. In a phase II trial, cemiplimab was evaluated in advanced NSCLC patients who were refractory or have progressed to at least first-line therapy. Twenty-one patients were enrolled. Grade 3 TEAEs occurred, including pneumonitis, diabetic ketoacidosis, and nephritis. The ORR was 28.6% (6/21) and DCR was 57.1% (12/21) [91]. A phase I study to evaluate a combination of cemiplimab plus REGN4659 (anti-CTLA-4) in advanced or metastatic NSCLC patient is ongoing, results not available yet.

JS001 is a humanized IgG4 anti-PD-1 antibody. In a phase I study, JS001 monotherapy was evaluated in patients with advanced or recurrent malignancies. Thirty-three patients were enrolled and the results indicate that it is well tolerated. No DLT was reported. Most TEAEs were grade 1–2. The ORR was 48.5% (16/33) and DCR was 70% (28/33). Two NSCLC patients achieved PR [92].

In a phase I/II study, spartalizumab (PDR001), an anti-PD1 monoclonal antibody was shown to have efficacy in advanced melanoma and NSCLC. The ORR was 9% (11/118) in NSCLC patients [93]. PDR001 in combination with platinum-doublet was evaluated in PD-L1 metastatic NSCLC patients; results are not yet available [94].

Camrelizumab (SHR-1210) is another PD-L1 monoclonal antibody. It is approved in China for the treatment of relapsed or refractory classical Hodgkin lymphoma. In a phase I/II study, camrelizumab plus apatinib was evaluated in advanced NSCLC patients for second-line treatment. Ninety-six patients with non-squamous NSCLC and wide type for EGFR and ALK were enrolled. Efficacy was evaluated in 91 patients. The ORR was 29.7% and DCR was 81.3%. Grade 3 and 4 TEAEs occurred in 56.2% of patients and the most commonly reported AEs were hypertension, hand-foot syndrome, proteinuria, gamma-glutamyl transferase increase, abnormal hepatic function, and alkaline phosphatase increase [95].

### Siglec-15 antibody

Siglec-15 is an immunoglobulin-like protein that can be upregulated in many human cancers. Siglec-15 expression works as a critical immune suppressor and is mutually exclusive to PD-L1.

NC 318 is a monoclonal antibody targeting Siglec-15 to normalize immune system. In a phase I study, 13 NSCLC patients who had prior treatments and PD-L1 refractory were enrolled and received NC 318 at different doses. Two patients achieved response with one CR and one PR. NC 318 was safe and well tolerated in 49 patients with advanced solid tumors. The dose-limiting toxicity was a grade 3 pneumonitis in the 1600 mg cohort [96].

### T cell therapy

MAGE-A10 is expressed in 10-50% of NSCLC, urothelial, melanoma, and head and neck cancer. Affinity-enhanced autologous MAGE-A10^c796^ T cells were evaluated in patients with these advanced cancers that had progressed after at least one line of therapy. In a phase 1 trial, these first-in-human T cells were given at 0.1 × 10^9^ to 8 patients. Grade 3 AEs were observed in 2 patients including pancytopenia and hyponatremia. One DLT of cytokine release syndrome was observed. No efficacy data is available yet [97].

### Autologous tumor-infiltrating lymphocytes

Tumor-infiltrating lymphocyte (TIL) therapies are a form of adoptive cell transfer (ACT) immunotherapy which has showed durable response in tumors with high mutation burdens. LN-144/LN-145 is a preparation of TIL extracted from surgically resected tumors, followed by culture with IL-2. LN-144/LN-145 is then infused back to the patient. A phase 2 study is ongoing to assess the efficacy and safety of LN-144/LN-145 alone and in combination with pembrolizumab in patients with advanced or metastatic solid tumors. One of the cohorts is for NSCLC patients who have received up 3 lines of prior therapy. No results are available yet. LN-144 reported to achieve 38% (21/55) ORR in metastatic melanoma patients who had 3.1 mean prior therapies. LN-145 induced 44% (12/27) ORR in patients with advanced cervical cancer who had at least 1 prior line of chemotherapy (NCT03645928) [98, 99].

### Adenosine 2a receptor (A_2a_R) antagonist

Adenosine signaling through A2a receptor on immune cells may have immunosuppressive effects. NIR178 is an oral A_2a_R antagonist that selectively inhibits A_2a_R and may reactivate T cell-mediated anti-tumor immune response. A phase I/II study evaluated NIR178 in pretreated NSCLC patients. Twenty-four patients were enrolled in the study. The most commonly reported AEs were nausea, fatigue, dyspnea, vomiting, and chest pain. Grade 3 drug-related AEs reported include pneumonitis (8%) and nausea (4%). No grade 4 AEs were reported. Efficacy was evaluated in 17 patients. One had CR, 1 PR, and 2 SD [100].

AB928 is a selective A_2a_R/A_2b_R antagonist. AB928 was evaluated in a phase 1 trial in combination with chemotherapy or AB122 (anti-PD-1) in patients with advanced tumors.

Nine patients were enrolled and six patients received AB928 and AB122. AB928 combination therapy was well tolerated. There were two evaluable patients and both had SD [101].

A phase I study to evaluate PBF-1129 (A2aR antagonist) in metastatic NSCLC is ongoing, results not yet available (NCT03274479).

### Anti-IL-8 monoclonal antibody

Interleukin 8 (IL-8) has been suggested to promote immune escape. BMS-986253 is a human monoclonal antibody that binds and inhibits IL-8. A phase I trial showed BMS-986253 monotherapy in metastatic or unresectable solid tumors was well tolerated in 15 patients and MTD was not reached at 32 mg/kg. TEAEs occurred in 5 patients and most were grade 1 with the exception of grade 2 fatigue, hypophosphatemia, and hypersomnia in 2 patients. Eleven (73%) patients achieved SD [102].

High levels of serum IL-8 are associated with poor outcome in NSCLC and decreases of IL-8 may be associated with response in NSCLC patients treated with nivolumab. A phase 1b/2 study of nivolumab in combination with BMS-986253, in a biomarker-enriched population of patients with advanced cancer is ongoing; results are not yet available [103].

### CD40 agonist

CD40 is an immune-activating TNF receptor. APX005M is a humanized monoclonal antibody binds to CD40 and activates antigen-presenting cells leading to stimulation of cancer-specific T cell responses.

In a phase I/II trial, APX005M plus nivolumab was evaluated in metastatic melanoma or NSCLC patients. One of 4 NSCLC immunotherapy naïve patients, had confirmed CR and 2 had SD [104].

SEA-CD40 is a non-fucosylated CD40 agonist that was evaluated in a phase I study in relapsed/refractory metastatic solid tumors. Forty-eight patients enrolled in the study and SEA-CD40 monotherapy was found to have a tolerable safety profile. DLT (infusion-related reactions) occurred in 5 patients. Common TEAEs were infusion-related reactions, chills, fatigue, nausea, vomiting, dyspnea, and headache. Efficacy was evaluated in 32 patients 1 had PR (basal cell carcinoma) and 10 had SD with DCR 32% [105].

### CD122-biased agonist

NKTR-214 is a CD122 biased agonist. It binds to CD-122, a subunit of IL-2 receptor on T and NK cells. NKTR-214 promotes the activation and proliferation of CD8^+^ T cells and NK cells. It also increases expression of PD-1 protein on T cells and PD-L1 on cancer cells. Thus, NKTR-214 may have potential synergistic effects with anti-PD-1 or anti-PD-L1 inhibitors against cancer.

In a phase I/II (PIVOT) trial, NKTR plus nivolumab was evaluated in patients with advanced solid cancers. The ORR was 50% and DCR was 67% in 6 NSCLC patients. Commonly reported TEAEs included flu-like symptoms, fatigue, rash, and pruritus [106].

A phase I/II trial of NKTR-214 combined with pembrolizumab or atezolizumab in patients with advanced solid tumors is ongoing, and results are not yet available [107].

### Cytokine therapy

ALT-803 is an IL-13 superagonist. In a phase I/II trial, ALT-803 was combined with nivolumab to treat patients with metastatic NSCLC. Among patients in phase Ib, who had PD1 immunotherapy relapsed or refractory tumors, the DCR was 91% (10 of 11), 27% (3 patients) had PR and 64% (7 patients) had SD. In 10 patients with PD-L1 negative tumors, the DCR was 70% and 30% had PR [108].

### Indoleamine 2,3-dioxygenase (IDO) pathway

Indoleamine 2,3-dehydrogenase (IDO) is an enzyme that catalyzes tryptophan into kynurenine. This enzyme is overexpressed in different malignancies. IDO causes depletion of tryptophan in the tumor microenvironment and tumor-draining lymph nodes thus inhibiting the function of immune effector cells.

IO102 is a second-generation immune-modulatory vaccine with a single IDO-derived peptide that activates CD8 T cells to kill IDO-expressing tumor cells and attacks immune suppressive cells. In a phase I study, the first generation IDO vaccine IO101 showed anti-tumor activity in pretreated advanced NSCLC. A phase I/II trial to evaluate IO102 in combination with pembrolizumab with or without chemotherapy as a first-line treatment in metastatic NSCLC patients is ongoing; results are not yet available [109, 110].

A phase I/II trial evaluated the IDO1 inhibitor, Epacadostat in combination with pembrolizumab in advanced NSCLC patients who have received platinum-based chemotherapy but no prior immunotherapy. Seventy patients were enrolled. The ORR was 29% (20/70) and the DCR was 50% (35/70). Grade 3 and 4TEAEs were reported in 27% of patients [111].

### Leukemia inhibitory factor (LIF) monoclonal antibody

LIF is expressed in multiple cancers and correlates with poor prognosis. Blocking LIF in mouse models has shown decreased tumor growth. A phase I study of MSC-1, a humanized anti-LIF monoclonal antibody in patients with advanced solid tumors is ongoing and results are not yet available [112].

### RORγ agonist

LYC-55716 is oral small-molecule agonist of retinoic acid receptor-related orphan receptor γ (RORγ). In a preclinical model, RORγ agonist decreases expression of PD-1 and other co-inhibitory receptors. RORγ and PD-1/PD-L1 inhibitors work synergistically. A phase 1b trial to evaluate safety and efficacy of RORγ agonist LYC-55716 in combination with pembrolizumab in metastatic NSCLC is ongoing; results are not yet available [113].

### SIRPα-CD47 immune checkpoint blockade

CD47 is expressed in all normal cells but overexpressed in tumor cells and it functions as an immune checkpoint in cancer. Signal-regulatory protein (SIRP) α is an inhibitory receptor expressed in myeloid cells. When CD47 in tumor cells binds to SIRPα in myeloid cells, it leads to suppression of tumor cell phagocytosis and other innate immune functions.

AXL148 is a fusion protein with high affinity for the CD47 binding domains in SIRPα that are linked to an inactive Fc region of human immunoglobulin. AXL148 has much higher affinity to bind to CD47 than the natural SIRPα. In a phase I study, ALX148 in combination with pembrolizumab or trastuzumab was evaluated in patients with advanced malignancy with checkpoint inhibitor relapsed or refractory diseases. A total of 108 patients were enrolled. Fifty NSCLC patients received AXL148+pembrolizumab. Commonly reported TEAEs were fatigue, AST increase, ALT increase, anemia, and platelets decrease. Efficacy was evaluated in 23 patients; 1 had PR and 8 had SD with DCR 39.1% [114].

### Anti-TIM-3 antibody and anti-LAG-3 antibody

T cell immunoglobulin and mucin containing protein-3 (TIM-3) is another immune checkpoint expressed on CD8 T cells that could inhibit cancer immunity. Lymphocyte activation of gene-3 (LAG-3) is a co-inhibitory receptor that is frequently co-expressed with PD-1 in tumor-infiltrating lymphocytes. LAG-3 is associated with impaired T cell function and with the combined inhibition of TIM3, PD-1, and LAG-3 may also increase anti-tumor activity and overcome the resistance to immunotherapy.

MK-4280 is a humanized immunoglobulin G4 anti–LAG-3 antibody. In a phase I/II trial, MK-4280 in combination with pembrolizumab was evaluated in metastatic solid tumor patients who have failed the standard treatment. The ORR was 27% and DCR was 40% in 15 patients [115].

TSR-022 is a selective Anti-TIM-3 antibody. In the phase I/II AMBER study, TSR-022 was evaluated as monotherapy or in combination with TSR-042 (anti-PD-1) and TSR-033 (anti-LGA-3) in advanced solid tumors. In the dose-expansion portion of the AMBER study, thirty-nine advanced NSCLC patients who had received prior anti-PD-1/PD-L1 treatment were enrolled to receive TSR-022 and TSR-042. Efficacy was evaluated in 20 patients who received TSR-022 at 300 mg dose; 3 achieved PR and 8 had SD. ORR was 33.3% (4/12) and DCR was 75% (9/12) in 12 PD-L1 ≥ 1% patients [116].

Eftilagimod alpha (Efti, IMP321) is a soluble LAG-3 fusion protein binds to major histocompatibility complex (MHC) class II and activates antigen-presenting cells. Efti has potential synergy with other therapeutic agents. In a phase II (TACTI-002) study, Efti plus pembrolizumab were given to 48 patients with metastatic NSCLC or head and neck carcinoma. TEAEs occurred in > 10% of patients and 2 TEAEs leading to treatment discontinuation: 1 with grade 4 hepatitis and another with grade 3 diarrhea. No new TEAEs. In part A of the study, 17 metastatic NSCLC patients received Efti+pembrolizumab as first-line treatment. ORR was 47% regardless of PD-L1 status. In part C of the study, ORR was 33% (6/18) in metastatic head and neck patients second-line treatment [117].

### Single domain antibody conjugate

L-DOS47 is a urease immune-conjugate. AFAFIKL2 (L) is a unique single domain antibody derived from liama antibody fragments. Urease (DOS47) is a microenvironment modifier and therapeutic agent. The AFFIKL2 antibody targets to deliver the enzyme and the urease enzyme convert urea into ammonia and increase the local pH level. In a phase I/II L-DOS47 monotherapy was evaluated for advanced non-squamous NSCLC patients, fifty-five patients were enrolled in sixteen cohorts. L-DOS47 monotherapy is well tolerated at dose levels up to 13.55 μg/kg. No CR or PR reported. Efficacy was evaluable in 32 patients who had SD, 13 had a decrease in the sum of diameters of target lesions [118]. Phase I/II studies to evaluate L-DOS47 in combination with cisplatin/vinorelbine (NCT03891173) in lung adenocarcinoma, and in combination with carboplatin/pemetrexed (NCT02309892) in recurrent or metastatic non-squamous NSCLC are ongoing. Results are not yet available yet.

### Vaccines

CV301 is a poxviral-based vaccine that could potentially increase the clinical benefit in combination with pembrolizumab. A phase 1 trial of CV301 has been completed and results indicate that there is no DLT. A phase 1b trial to evaluate combination of CV301 and pembrolizumab in NSCLC patients is ongoing, results are not yet available [119].

Vaccine BI 1361849 (CV9202) has 6 mRNA encoding for selected tumor-associated antigens: MUC1, survivin, NY-ESO-1, 5 T4, MAGE-C2, and MAGE-C1. In a phase Ib study, BI 1361849 combined with local radiation were given to 26 stage IV NSCLC patients with progression of disease after standard first-line therapy. Common BI 1361849-related adverse events are injection site reactions and flu-like symptoms. The majority (84%) of the patients have shown an increased BI 1361849 antigen-specific immune response. One patient had PR in combination with pemetrexed maintenance, and 46.2% had SD [120].

A phase I/II trial to evaluate BI 1361849 in combination with durvalumab or durvalumab + tremelimumab in patients with metastatic NSCLC is ongoing, results not available yet. This study combines active and passive immunotherapy [121].

Dendritic cell vaccine (DCVAC) can present tumor antigen to induce a durable immune response. In a phase II study, DCVAC in combination with standard chemotherapy was evaluated in metastatic NSCLC patients. When compared to chemotherapy alone, DCACV + chemotherapy improved the ORR (45% vs 34%) and median OS (15.5 months vs 11.8 months) [122].

Universal cancer peptide-based vaccine (UCPVax) is a vaccine directed towards universal cancer peptide telomerase to activate CD4 helper T lymphocytes. A phase I/II trial to evaluate UCPVAx in patients with metastatic NSCLC is ongoing; results are not yet available (NCT02818426). DNA damage results in cancer neoantigens. Neoantigens may serve as targets for tumor-directed immune responses. The combination of immunotherapy and chemotherapy reduces early progression and may modulate the tumor microenvironment, which has been shown to improve survival over chemotherapy alone in patients with NSCLC. Neoantigen vaccines in combination with immunotherapy and chemotherapy may induce T cell reactivity and expand T cell response against neoantigens.

NEO-PV-01 is a personal neoantigen vaccine customized for the molecular profile of each individual’s tumor. NEO-PV-01 in combination with nivolumab induced broad de novo neoantigen-specific immune response in 10 metastatic melanoma patients [123].

A phase 1 clinical trial to evaluate the safety and response of NEO-PV-01 in combination with pembrolizumab plus chemotherapy in patients with advanced or metastatic non-squamous non-small cell lung cancer is ongoing; results are not yet available [124].

EVAX-01-CAF09b is a personalized neoantigen vaccine with up to 15 peptides derived from somatic mutation of patient’s cancer. A phase I study to evaluate the vaccine in combination with anti-PD-1/anti-PD-L1 inhibitor in patients with advanced NSCLC, melanoma, and kidney cancer is ongoing, no results available (NCT03715985).

ADXS-NEO is a live, attenuated *Listeria monocytogenes* (Lm) immunotherapy, using ≥ 20 personal neoantigens and a truncated fragment of listeriolysin O (tLLO). Two patients experienced DLTs at 1X109 CFU dose level. Dose at 1X108 CFU was found to be safe and tolerated by one patient. ADXS-NEO is able to induce specific CD8+ T cells that recognized 90% of the 40 cancer neoantigen targets inserted in the Lm bacteria. The study is recruiting patients with advanced solid tumors for ADXS-NEO monotherapy or in combination with pembrolizumab [125].

### HDAC inhibitor and immunotherapy combination

Histone deacetylase (HDAC) inhibitor may upregulate major histocompatibility protein (MHC) and PD-L1 thus enhancing the efficacy of immunotherapy.

ACY-241 is a HDAC6 inhibitor. In a phase Ib study, ACY-241 in combination with nivolumab was tested in platinum-treated and immunotherapy naïve advanced NSCLC patients. Eighteen patients were treated. Recommended phase 2 dose is 360 mg. Efficacy was evaluated in 13 patients, 8 had clinical benefit: 1 CR, 3 PR, 1 unconfirmed PR, and 3 SD. Commonly reported TEAEs included fatigue, arthralgia, and cough [126].

Entinostat is a HDAC1 and HDAC3 inhibitor. Entinostat plus pembrolizumab was evaluated in advanced NSCLC patients who had progressed on anti-PD-1/PD-L1 therapy. Fifty-seven patients were enrolled, and 5 patients achieved a confirmed PR. The ORR was 9%. The median duration of response is 4.2 months. The most common immune-related AEs were fatigue, anemia, decreased appetite, and diarrhea. Grade 3/4-related AEs occurred in 35.1% of patients [127].

Vorinostat is a HDAC inhibitor acts on class I, II, and IV of histone deacetylase. Vorinostat is FDA-approved for the treatment of cutaneous T cell lymphoma. In a phase I/Ib trial, vorinostat plus pembrolizumab was evaluated in patients with metastatic NSCLC that had been treated with or without immune checkpoint inhibitors (ICIs). Fourteen patients were enrolled to phase I and 20 patients were enrolled to phase Ib. The recommended phase 2 dose was 200 mg of pembrolizumab and 300 mg of vorinostat. Efficacy was evaluable in 30 patients, 6 were ICIs naïve and 24 ICIs-pretreated. The ORR was 13% (4/20) and SD was 53% (16/30). In ICI-pretreated phase Ib cohort, 2 patients achieved PR and 10 had SD. The most commonly reported grade 3 AEs were myalgia, anemia, and diarrhea [128].

### BET inhibitor

PLX51107 is a small molecule that exhibits interactions mediated by the four BET family proteins. PLX51107 is a novel drug targeting epigenetic approach. Phase Ib/IIa study results indicate that the MTD of PLX51107 is between 200 and 300 mg. Three patients had grade 3 TEAEs. One patient with NSCLC achieved SD [129].

### SMAC mimetics

Debio 1143 is an oral antagonist of IAPs (inhibitor of apoptosis proteins) increased PD-1/PD-L1 expression and tumor-infiltrating lymphocytes thus synergizes with PD1/PDL1 inhibitors in preclinical models. Debio 1143 promotes apoptosis of cancer cells by mimicking the activity of second mitochondria-derived activator of caspase (SMAC).

Sixteen advanced solid tumors patients were treated with Debio 1143 plus avelumab In a phase I study. One patient had DLT at Debio 1143 250 mg/day dose (grade 3 ALT/AST increase). Most TEAEs were grade 1 or 2. Efficacy was evaluable in 15 patients, one had PR (NSCLC) and five had SD. Two other NSCLC patients had > 15% tumor shrinkage [130].

## Conclusions

Significant advances have been made in the treatment of advanced and metastatic NSCLC. We have seen median survival improved from 4 to 6 months with best supportive care then progress to 8–17 months with chemotherapy. Currently, with targeted therapy and immunotherapy, the median overall survival rate for this devastating disease is estimated to be 18–36 months and some have long-term survival. The 5-year overall survival was about 4–6% percent and now the 5-year survival rate when treated with immunotherapy is 15–20%. The median OS is more than 3 years for EGFR mutant patients with EGFR TKIs and patients with ALK rearrangements also enjoy long-term survival with multiple lines of ALK TKIs. However, targetable mutations are not present in all patients. Patients that are on targeted therapy or immunotherapy will eventually develop treatment resistance. Identifying new targets, understanding the resistance mechanisms and developing novel agents or strategies to overcome the resistance are needed. Although immunotherapy can provide durable response and long-term survival in some patients, a significant percentage of patients may not benefit from it and some may develop super progression after initiation of immunotherapy. Efforts to find reliable biomarkers to guide the selection of patients who may benefit from immunotherapy are undergoing.

**Table 6:** Summary of phase I/II clinical trials for advanced/metastatic NSCLC

Drug Class	Drug	Mechanism of action	Study design	Clinical trial	Phase	Type of cancer	Status
Targeted therapy	Ensartinib	ALK inhibitor	Monotherapy	NCT03215693	I	NSCLC	Recruiting
	Ensartinib	ALK inhibitor	Monotherapy	NCT01625234	I/II	NSCLC	Recruiting
	Repotrectinib (TPX-0005)	ALK/ROS1/NTRK inhibitor	Monotherapy	NCT03093116	I	ALK/ROS1/NTRK+ solid tumors	Recruiting
	M6620 (VX-970)	ATR inhibitor	Combination with gemcitabine	NCT02157792	I	NSCLC	Active, not recruiting
	Bemcentinib	AXL inhibitor	Combination with docetaxel	NCT02922777	I/II	NSCLC	Recruiting
	Bemcentinib	AXL inhibitor	Combination with pembrolizumab	NCT03184571	II	NSCLC	Recruiting
	TP-0903	AXL inhibitor	Monotherapy	NCT02729298	I	Advanced solid tumors	Recruiting
	Nazartinib	EGFR	Monotherapy	NCT02108964	II	EGFRmut solid tumors	Active, not recruiting
	BPI-7711	EGFR 790 M inhibitor	Monotherapy	NCT03386955	I	NSCLC	Recruiting
	TAK-788	EGFR and HER2 TKI including exon 20 insertions	Monotherapy	NCT02716116	I/II	NSCLC	Recruiting
	YH25448	EGFR and T790M	Monotherapy	NCT03046992	I/II	NSCLC	Recruiting
	HS-10296	EGFR inhibitor	Monotherapy	NCT02981108	I/II	NSCLC	Recruiting
	SPH1188-11	EGFR inhibitor	Monotherapy	NCT03231475	I	NSCLC	Recruiting
	Poziotinib	EGFR or HER2 exon 20 inhibitor	Monotherapy	NCT03318939	II	NSCLC	Recruiting
	Pyrotinib	HER1/HER2/HER4 inhibitor	Monotherapy	NCT02834936	I	NSCLC	Unknown
	AMG510	KRAS G21C inhibitor	Monotherapy	NCT03600883	I/II	KRAS g12C mutation solid tumors	Recruiting
	MRTX849	KRAS G21C inhibitor	Monotherapy	NCT03785249	I/II	KRAS g12C mutation solid tumors	Recruiting
	Capmatinib (INC280)	MET inhibitor	Monotherapy and in combination with gefitinib	NCT02468661	I	NSCLC	Recruiting
	Tepotinib	MET inhibitor	Monotherapy	NCT02864992	II	NSCLC	Recruiting
	LOXO-292	RET inhibitor	Monotherapy	NCT03157128	I	RET (+) cancers	Recruiting
	DS-6051b	ROS1/NTRK inhibitor	Monotherapy	NCT02279433	I	ROS1 and NTRK Advanced carcinoma	Active, not recruiting
	MP0250	VEGF and HGF neutralizing DARPIN molecule	Monotherapy	NCT02194426	I	Advanced solid tumors	Completed
Antibody-drug conjugate	PF-7020	ADC targeting protein tyrosine kinase 7 (PTK7)	Monotherapy	NCT02222922	I	Advanced solid tumors	Active, not recruiting
	BA3011	Anti-AXL antibody	Monotherapy	NCT03425279	I/II	Advanced solid tumors	Recruiting
	EnaV	Anti-AXL antibody	Monotherapy	NCT02988817	I/II	Advanced solid tumor	Recruiting
	U3-1403	Anti-HER2 antibody-drug conjugate	Monotherapy	NCT03260491	I	NSCLC	Recruiting
	Anetumab	Anti-mesothelin IgG1 antibody conjugated to the maytansinoid tubulin inhibitor DM4	Monotherapy or in combination	NCT03102320	I	Advanced solid tumors	Recruiting
	BT1718	Bicycle drug conjugate, binds MT1-MMP and linked to DM1	Monotherapy	NCT03486730	I/II	Advanced solid tumors	Recruiting
	JNJ-372	EGFR-cMet bispecific antibody	Monotherapy	NCT02609776	I	NSCLC	Recruiting
	XMT-1522	HER-2 Targeting antibody-drug conjugate	Monotherapy	NCT02952729	I	Her 2 expressing breast, lung, and gastric cancer	Active, not recruiting
	DS-1062a	TROP2-targeting antibody-drug conjugate	Monotherapy	NCT03401385	I	Advanced solid tumors	Recruiting
	DS-8201a	HER-2 Targeting antibody-drug conjugate	Monotherapy	NCT02564900	I	HER2 expression solid tumors	Active, not recruiting
Cell therapy	MAGE-A10 T cell therapy	Affinity-enhanced T cells against MAGE A10 antigen	Monotherapy	NCT02592577/NCT02989064	I	Advanced solid tumors	Recruiting
	CRISP/Cas9-mediated knockout of PD-1 gene	T lymphocytes with PD-1 gene knockout	Monotherapy	NCT02793856	I	NSCLC	Active, not recruiting
Cytokine therapy	ALT-803	IL-15 superagonist	Combination with nivolumab	NCT02523469	I/II	NSCLC	Active, not recruiting
Immuno-Conjugate	L-DOS47	Urease immuno-conjugate	Combination with cisplatin+vinorolbine	NCT03891173	I/II	NSCLC	Recruiting
Immuno-Conjugate	L-DOS47	Urease immuno-conjugate	Combination with carbo+pemetrexed	NCT02309892	I/II	NSCLC	Recruiting
Immunotherapy	NIR178	A2AR antagonist	Monotherapy	NCT02403193	I/II	NSCLC	Recruiting
	PBF-1129	Anti-A2aR	Monotherapy	NCT03274479	I	NSCLC	Recruiting
	AB928 and AB122	Anti-A2aR and A2bR, Anti-PD-1	Monotherapy and combination therapies	NCT03846310	I	NSCLC	Recruiting
	REGN4659	Anti-CTLA-4	Combination with cemiplimab	NCT03580694	I	NSCLC	Recruiting
	BMS-986253 (HuMax-IL8)	Anti-IL-8 monoclonal antibody	Monotherapy	NCT02536469	I	Advanced solid tumors	Completed
	MSC-1	Anti-leukemia inhibitory factor(LIF) antibody	Monotherapy	NCT03490669	I	Advanced solid tumors	Active, not recruiting
	Cemiplimab	Anti-PD-1	Monotherapy	NCT02383212	I	Advanced malignancies	Active, not recruiting
	JS001	Anti-PD-1	Monotherapy	NCT02836834	I	Advanced or recurrent malignancies	Active, not recruiting
	PDR001	Anti-PD-1	Combination with platinum doublet	NCT03064854	I	NSCLC	Recruiting
	SHR-1210	Anti-PD-1	Combination with apatinib	NCT03083041	I/II	NSCLC	Unknown
	Tislelizumab	Anti-PD-1	Combination with chemotherapy	NCT03432598	II	NSCLC	Recruiting
	NC318	Siglec-15 antibody	Monotherapy	NCT03665285	I	Advanced solid tumors	Recruiting
	TSR-022,TSR 042, TSR033	Anti-TIM-3, Anti-PD-1, and anti-LGA3	Monotherapy and in Combination	NCT02817633	I	Advanced tumor	Recruiting
	Eftilagimod alpha	LAG-3 fusion protein	Combination with pembrolizumab	NCT03625323	II	NSCLC and HNSCC	Recruiting
	NKTR-214	CD122-biased agonist	Combination with nivolumab	NCT02983045	I/II	Advanced cancers	Recruiting
	NKTR-214	CD122-Biased Cytokine	Combination with pembrolizumab or atezolizumab	NCT03138889	I	NSCLC	Recruiting
	APX005M	CD40 agonistic antibody	Combination with nivolumab	NCT03123783	I/II	NSCLC	Recruiting
	SEA-CD40	CD40 Antibody	Monotherapy and in Combination with pembrolizumab	NCT02376699	I	Relapsed/refractory metastatic solid tumors	Recruiting
	ALX148	CD47 blocker	Monotherapy and combination with pembrolizumab, trastuzumab, or rituximab	NCT03013218	I	Advanced solid tumors and lymphoma	Recruiting
	IO102	IDO vaccine	Combination with pembrolizumab with or without chemotherapy	NCT03562871	I/II	NSCLC	Recruiting
	Epacadostat	IDO1 inhibitor	Combination with pembrolizumab	NCT02178722	I/II	Solid tumors	Active, not recruiting
	BI1361849	mRNA vaccine	Combination with durvalumab and/or tremelimumab	NCT03164772	I/II	NSCLC	Recruiting
	EVAX-01-CAF09b	Neo-antigen vaccine	Montherapy	NCT03715985	I	Advanced solid tumors	Recruiting
	NEO-PV-01	Personal neoantigen vaccine	Combination with pembrolizumab +chemotherapy	NCT03380871	I	NSCLC	Active, not Recruiting
	ADXS-NEO	Personalized neoantigen-listeria vaccine	Monotherapy and combination with pembrolizumab	NCT03265080	I	Advanced tumor	Recruiting
	LYC-55716	RORγ agonist	Combination with pembrolizumab	NCT03396497	I	NSCLC	Active, not recruiting
	LN-144/LN-145	Tumor-infiltrating lymphocytes	Monotherapy and combination with pembrolizumab	NCT03645928	II	NSCLC	Recruiting
	CV301	Vaccine	Combination with pembrolizumab	NCT02840994	I	NSCLC	Active, not recruiting
	DCVAC	Vaccine	Combination with chemotherapy	NCT02470468	I/II	NSCLC	Active, not recruiting
	UCPVax	Vaccine	Montherapy	NCT02818426	I/II	NSCLC	Recruiting
	Debio 1143	Smac mimetics	Combination with avelumab	NCT03270176	I	NSCLC	Recruiting
Tumor epigenetics	ACY 241	HDAC inhibitors	Combination with nivolumab	NCT02635061	I	NSCLC	Recruiting
	PLX51107	BET inhibitor	Monotherapy	NCT02683395	Ib/IIa	advanced hematological and solid tumors	Terminated
	Entinostat	HDAC inhibitors	Combination with pembrolizumab	NCT02437136	I/II	NSCLC	Active, not recruiting
	Vorinostat	HDAC inhibitors	Combination with pembrolizumab	NCT02638090	I/II	NSCLC	Recruiting

In the past decade, the FDA has approved multiple targeted agents for patients who have actionable mutations such as EGFR, ALK, ROS1, BRAF, and NTRK. Promising targeted agents for KRAS G12C, RET, MET, and AXL as well as other mutations are being currently investigated and could receive approval in the near future. For patients who do not have actionable mutations, the best alternatives are immunotherapy alone if PD-L1 expression is more than 50% or in combination with chemotherapy if PD-L1 with low expression. The exploration of immunotherapy in advanced/metastatic NSCLC has been beyond anti-PD-1/PD-L1 and CTLA pathway. Immunotherapy to stimulate immune response through other pathways such as IDO, TIM, LAG-3, CD40, and CD122 are encouraging. Innovative treatments such as neoantigen vaccine and antibody-drug conjugate are also being tested in this patient population (Table 6).

The study and treatment innovations in advanced and metastatic NSCLC are probably one of the most active and promising in the field of metastatic solid tumors.

## Data Availability

All data and materials used for this study are included in this article.

## Abbreviations

DCR: Disease control rate
FDA: Food and Drug Administration
NSCLC: Non-small cell lung cancer
ORR: Overall response rare
OS: Overall survival
PD: Progressive disease
PR: Partial response
SD: Stable disease
TEAEs: Treatment-emergent adverse events
TPS: Tumor proportion score
